# Care of the Hands

**Published:** 1912-03-15

**Authors:** Ralph St. J. Perry


### CARE OF THE HANDS.

BY RALPH ST. J. PERRY, M.D.

During the years that have elapsed since Lister promulgated his antiseptic edict of cleanliness, much has been written upon the rendering of the hands aseptic, so much, in fact, that in the thrusting of the aseptic "mit" into the lime-light many have overlooked one of the most potent factors in medical economics—the ordinary care of the doctor's hands.

The doctor's *hands should be clean, dry, warm, smooth, and soft.* Regardless of the social classification of his clientele or of his own standing in the community—whether as a general practitioner or a specialist—whenever he gives the grasp of friendship or he makes a physical examination his friends, patients, as well as innocent bystanders should be witness to the fact that his hands possess those five qualities enumerated, namely, cleanliness, dryness, warmth, smoothness, softness. This possession does not in any way imply weakness or effeminateness, it merely indicates compliance with a business necessity.

### CLEANLINESS OF THE HANDS.

The conditions of our mundane existence are such that the hand becomes the lodging place for more or less dirt—matter out of place; and because of the many peculiar requirements of our professional work, we come in contact with many kinds of dirt not within the ordinary manual affinity of others.

In cleansing the hands, it is best to use warm soft water, if possible. If not obtainable, use hard water which has been boiled for half an hour or to which a little ammonia or borax has been added. The solvent effect of the water is more fully secured if the water be warm. Soap is a necessity to the laving, and after some forty years of saponaceous deterging I am still adhering to the old reliable coconut-oil soap of my youth and concomitant dirtiness. The coconut-oil makes a purely vegetable soap, and when properly made it is hard, white, almost odorless, and will form a good, cleansing lather either in hard or soft, warm or cold water. It is not expensive and can be bought anywhere.

Various antiseptic soaps are on the market, but such are not needed for ordinary cleansing. Soft and liquit soaps, while very nice for your office wash-stand, can not well be carried about on one's trips. Always carry your own cake of soap; it is safer and cleaner then the promiscuously used piece commonly found away from home. In the farm home you too often are offered a chunk of laundry soap, which because of the rosin it contains, is not good for toilet purposes, this articles leaving the hands gummy and sticky. Soap leaves—sheets of tissue-paper coated with soap—are handy, when obtainable.

There are two best times when to wash the hands, namely, just before examining a patient, and just after. The frequent repetition of this practice is perfectly harmless, while the psychic effect upon the patient and others is beneficial.

### HOW TO WASH AND DRY THE HANDS.

In washing the hands do not merely dip them in water and wipe the dirt off on the towel; that is a poor way. Take off your coat, remove your cuffs, pull your shirt sleeves up to the elbows, undershirt ditto, then wash well up along the forearm. That little dark line of demarkation we so often see at the wrists is more an evidence of slovenliness than of uncleanliness. Wash the dirt all off into the water, leaving as little as possible to be wiped off on the towel.

The towel was intended for drying purposes, hence should be of an absorbent material and woven thick enough to be serviceable. Turkish and huck towels are better than crash or thin cotton stuff. The paper towel, which has been recently introduced, is better and cheaper than a cloth towel, which has to be laundered. As stated, the function of the towel is desiccative or dehydrative, and in carrying out this part, there should be a thorough and careful wiping at all joints and in the interdigital clefts. Imperfect drying leads to red and chapped hands, roughness of the skin, and eczema.

### THE FINGER-NAILS NEED CLOSE ATTENTION.

The physician who drives, rides a bicycle, runs an automobile or uses the cars, should keep his hands as clean as possible by wearing suitable gloves all the year around.

Another, and most important matter not to be overlooked in cleansing the hands is that the finger-nails require careful cleaning also. Use a nail-cleaner, your pen-knife blade, a toothpick, the chewed end of a match or any suitable extemporaneous tool, but, by all means, let people see the natural color of your projecting nail-tips. Not even if you have suffered bereavement should your melanotic emblem of mourning be shifted from your sleeve to the finger-nails.

I have in mind a surgeon who, some years ago, was on the highway to fame and fortune. He had acquired positions of honor and a good income. But in spite of his brilliant discourses and equally brilliant results (in some classes of work), he lost many patients by death. At first, as a nonresident admirer, I sympathized with him in what seemed to be his "hard luck." Then, one day, while I was discussing surgical affairs with a noncombative resident, the latter chanced to refer to our "unlucky" friend as "Old Dirty Fingernails." This I found was the sobriquet bestowed upon that surgeon in the inner circle of those thereabout who chirurge—and in an instant there came an enlightening flood of understanding.

### OFFENSIVE ODORS—HOW TO HANDLE THEM.

One of the idiosyncrasies of personal contact which the doctor meets is the odoriferous patient. The aroma may be that of pus, of feces, vaginal secretion, senile smegma, amniotic fluid or any other of the odorific secretions, and this effluvium may possess a tenacity and lastingness that maliciously defies your most frantic washings and scrubbings, irrespective of strenuous repititions. There may be a hyperesthesia of your Schneiderian membrane or a hypertrophy of your imagination as factors in the case, but to you the proboscial offense is a reality, and it may be no less so to others.

As a deodorizing treatment, I have found the following very effective: Wash the hands thoroughly in warm water, using coconut-oil soap freely to produce a good lather; then wash in a solution of cyanide of mercury, 1:1000. Dry the hands *thoroughly*, after which rub a few drops of pure balsam of Peru into the skin. This imparts a vanilla-like odor to the hands and effectually masks the offensive odor. If necessary to leave the office at once, a nongreasy toilet cream (made of casein) perfumed with oil of rose may be applied; but if possible, use the balsam of Peru, as it is not only disodorizing but healing and antiseptic as well.

The same method may be employed in overcoming the odor of iodoform, carbolic and lysol solutions, valerian or any other unpleasant-smelling drugs with which the hands may have come in contact; so, also, against the hippuric, hippofecal and generally manurial and similar odors which attach to the doctor who personally attends to his barn duties. As a counter or foil for the odor of automobile oil and machine grease it is A No. 1,—but I doubt if anyone who pays a high price to acquire such a luxury would be very anxious to get rid of that. However, the suggestionis offered.

### DETERGENT METHODS FOR SUNDRY SOILS AND STAINS.

Grease and grime, paint, tar, printers' ink, asphalt, and matters of this nature call for more than soap and water. A little kerosene, benzin, gasolin or oil of turpentine rubbed on the hands as a preliminary to the washing will soften—"cut," as is the saying—the adherent dirt, so that most of it can be wiped off with a rag or bit of soft paper. Then wash freely with the coconut-oil soap or a real good tar soap (I prefer "Grandpa's"), adding a few drops of oil of turpentine or kerosene *after* the lather has formed. (Remember the injunction "after"!) Such a procedure will leave the hands clean, white, and soft. Clean diligently around and under the finger-nails with a stiff-bristle brush. Any remaining odor can be covered up with a few drops of balsam of Peru.

A most excellent type of soap for assisting in the removal of grime, paint, and dirt-matter of a smudgy, adhesive nature are those containing an admixture of fine sand, rottenstone, pumice or marble dust. Originally introduced, years ago, as "hand sapolio," these detergent soaps are now a general commodity as "mechanics' soap," "pumo," and under various other seductive trade-names. Most of them are made with coconut-oil soap as a basis—hence are satisfactory.

### STAINS PRODUCED BY CHEMICAL AGENTS.

Chemical stains upon a doctor's hands, even if ocular evidence of profound laboratory research, are not always interpreted as such. When the hands have been stained by a strong alkaline solution, wash them with a diluted acid (nitric or acetic—1 part in 100 parts of water); then, immediately, thoroughly rub soap into the skin of the wet hand, using no water. The acid, decomposing the soap (sodium oleostearate) brings about a deposit of fatty acids in the skin, which counteract the effects of the alkali and tend to prevent the skin cracking which otherwise would follow.

Nitric acid, which many of us use so freely and carelessly stains the skin yellow, but this discoloration can be removed by wetting with water, rubbing with soap, then washing in water and scrubbing with mechanics' soap. If soreness follows this treatment, apply lanolin containing a little aristol or thymol iodide, or, if handy, an oil-solution of the latter. The stains resulting from the other mineral acids generally can be removed in a like manner.

Iodine stains can be bleached out by applying a 10-per-cent solution of sodium hyposulphite; those of corrosive-sublimate solutions, by repeated washings or rubbings with a fairly strong solution of table-salt followed by inunctions with lanolin. Permanganate stains vanish upon application of a not too strong a solution of oxalic acid which has been slightly acidulated with sulphuric acid.

Battery fluid, i. e., the bichromate of potassium solution, stains the skin yellow; this, however, is removed by washing in a strong solution of sodium hyposulphite to which a little sulphuric acid has been added. Stains from some aniline dyes are removable by washing first in alcohol containing a little acetic acid and then rubbing with Javelle water (chlorinated-potassa solution, N. F.) or Labarraque's solution (chlorinated-soda solution, U. S. P.). The latter—now quite commonly also designated as Javelle water—is a preparation used by professional cleaners and scourers. Many formulas for it are in vogue (see U. S. Dispensatory), of which the following is one, though unusual: Sodium bicarbonate, 4 parts; water, 8 parts; chlorinated lime, 1 part. Dissolve the soda in the water, gently boil a few minutes, add the lime, allow to cool, then strain or filter. The boiling serves merely in facillitating precipitation of the calcium-carbonate magna, but it also dissipates the active gases.

Stains produced by berries, grass, walnut-husks, and in fact all stains due to vegetable juices can be removed by repeated application of this chlorinated water; likewise ink stains.

### DRYNESS AND WARMTH.

Supplemental to my previous remarks on keeping the hands dry, let me state that the hands, so far as possible, should be kept dry in summer, at which season the sweat-glands are prone to "work overtime." Some individuals are afflicted with excessive perspiration at all times, and these should be particularly careful to keep their hands dry. The moist hand is almost always a cold one, and a "cold, clammy" hand has a most depressing effect in business relations, especially with superstitious people and those of the original stock from the "ould sod" of the Emerald Isle. You may tell them as often and emphatically as you wish that "a cold hand means a warm heart," the chances are that they're "against ye."

A warm hand means a good capillary circulation and is usually an indication of good health. It is certainly an asset in the field of medicine and surgery when it becomes necessary to make manual or digital examinations.

### BEWARE THE COLD, CLAMMY HAND. GLOVES.

When you know your hand is cold (as you can readily test by applying it to your own cheek or throat), it can readily be warmed in a few minutes by holding it in a pan of warm water.

Prophylaxis, however, is far better than corrective measures. Keep your capillary circulation in good order, and be sure to wear warm gloves, according to the season and climate. In summer a thin cloth glove will answer; in the spring and fall a golf-glove, with a thin white cotton military dress-parade or a pall-bearer's glove inside of it, keeps the hand warm, dry, and free from the scratching of the unlined golf-glove; the white cotton glove being washed as often as required. Such a double glove has proved in actual service to be warmer than one of thick, heavy cloth.

In winter time, especially in the northern half of the

United States and in Canada, where the mercury runs low, it becomes necessary to resort to fur gloves or fleece-or fur-lined ones. During a score of years spent in driving over northwestern prairies, frost-nipped and blizzard-swept, I have worn all manner of fur and leather gloves and mittens, from sealskin to catskin, and there is no doubt in my mind that the warmest ones are those made from muskrat skin lined with fleece or with rabbit fur.

A winter-glove should fit loosely, as a tight glove compresses the digital arteries, shuts off the blood supply and really defeats its own purpose and makes the hand cold by depriving the fingers of their supply of blood-heat. A snug mitten is all right if one can drive with mittens, or if you have a driver; a too large mitten fails to conserve the heat and is cumbersome and awkward to manage.

### SMOOTHNESS.

A smooth condition of the hands not only makes them more presentable, but it also helps in the development of the tactile sense and affords fewer places for the lodgemnt and development of germs. Callosities from unusual work or of an occupational nature should be removed. Apply to the spots some salicylated flexible collodion; after a few hours an epidermal layer can be scraped away. Repeat as often as necessary, to get rid of the superfluous epidermis. This same treatment may be used for all varieties of warts.

Cracks due to chapped skin, exzema, and all other forms of skin disease incapacitate the physician for any form of surgical, obstetrical or gynecological work and should be cured as expeditiously as possible.

The finger-nails should be trimmed to shape, with well-smoothed and even edges. They may be polished or not, as the taste of the individual may incline. The nails are naturally of a smooth, satiny finish and pink color, and defects in finish and color can easily be overcome by the physician himself, or else by the manicurist. Hang-nails are an abomination in the eyes of the aseptic, hence should always be removed. Ganglions, wens, and any tumors that may develop are to be gotten rid of, for they are unsightly and, if neglected, serve as a suggestion to others that they can do likewise as to their own troubles—to your financial detriment.

### SOFTNESS.

A soft feel of the hands goes with that greatest of all qualities of the doctor—the gentle touch. The doctor who is rough in his work very often errs in diagnosis, for the reason that his roughness causes him to miss many points in an examination which are only perceptible to the easy, gentle pressure of the soft finger-tips. Handle a chestnut-bur gently and you feel all of its thousand spicules; grasp it roughly, and you feel them not. I have noticed, too—if I may here interject an economic note—that the surgeon who possesses this gentle touch is usually very successful in the financial touch.

Every man is called upon to do more or less rough manual work about the house, and it behooves him to see to it that his hands do not get rough. For this purpose he can use local applications of petrolatum, cold-cream, glycerite of tragacanth, coconut-oil, or any one of the score of efficient toilet creams and ointments known to the trade. They may be used plain, medicated or perfumed, as desired. It will be found in this matter, as in others, that there are idiosyncrasies, and what will quickly benefit one skin will irritate another, or have no effect upon it. Each individual must learn his own peculiarities by experience.

One of the little nick-nacks I have used for years with patients of this class is a putty-ball (fresh and of good quality), about the size of an orange, which the rough-handed person is instructed to manipulate and "work" for half an hour at a time. The exercise of the manipulation develops the muscles and fills out the thin, scrawny hands while the oil in the putty softens and cleanses the skin.—*Clinical Medicine.*

